# Spiking Patterns and Their Functional Implications in the Antennal Lobe of the Tobacco Hornworm *Manduca sexta*

**DOI:** 10.1371/journal.pone.0023382

**Published:** 2011-08-29

**Authors:** Hong Lei, Carolina E. Reisenman, Caroline H. Wilson, Prasad Gabbur, John G. Hildebrand

**Affiliations:** 1 Department of Neuroscience, University of Arizona, Tucson, Arizona, United States of America; 2 Department of Biology, University of Alaska, Anchorage, Alaska, United States of America; 3 Department of Computer Science, University of Arizona, Tucson, Arizona, United States of America; Center for Genomic Regulation, Spain

## Abstract

Bursting as well as tonic firing patterns have been described in various sensory systems. In the olfactory system, spontaneous bursts have been observed in neurons distributed across several synaptic levels, from the periphery, to the olfactory bulb (OB) and to the olfactory cortex. Several *in vitro* studies indicate that spontaneous firing patterns may be viewed as “fingerprints” of different types of neurons that exhibit distinct functions in the OB. It is still not known, however, if and how neuronal burstiness is correlated with the coding of natural olfactory stimuli. We thus conducted an *in vivo* study to probe this question in the OB equivalent structure of insects, the antennal lobe (AL) of the tobacco hornworm *Manduca sexta*. We found that in the moth's AL, both projection (output) neurons (PNs) and local interneurons (LNs) are spontaneously active, but PNs tend to produce spike bursts while LNs fire more regularly. In addition, we found that the burstiness of PNs is correlated with the strength of their responses to odor stimulation – the more bursting the stronger their responses to odors. Moreover, the burstiness of PNs was also positively correlated with the spontaneous firing rate of these neurons, and pharmacological reduction of bursting resulted in a decrease of the neurons' responsiveness. These results suggest that neuronal burstiness reflects a physiological state of these neurons that is directly linked to their response characteristics.

## Introduction

Neurons in various sensory systems produce action potentials in brief bursts of high frequency discharge as well as in tonic patterns [1]. In the olfactory system, the diversity of firing patterns have been observed in neurons distributed across several synaptic levels, including the periphery [2], the olfactory bulb (OB) [3]–[5] and the olfactory cortex [6]. At the OB level, one type of juxtaglomerular cells, the external tufted cells, intrinsically fire bursts of action potentials at theta frequency while other types of juxtaglomerular cells do not produce spike bursts spontaneously [3]. Similarly, the tufted cells of the external plexiform layer also fire rhythmic bursts of spikes. In contrast, the intrinsic GABAergic interneurons at this layer rarely fire any action potential spontaneously [7]. These *in vitro* observations suggest that spontaneous firing patterns are different in different types of neurons within the OB circuit. Despite that bursting patterns are commonly observed, it is largely unexplored if neuronal burstiness could be correlated with the function of encoding natural olfactory stimuli in a predictable fashion.

We thus conducted an *in vivo* study to probe this question in the OB equivalent structure of insects, the antennal lobe (AL), of the moth *Manduca sexta* [8]. As in their vertebrate counterparts, AL glomeruli are the sites where synaptic interactions occur among afferent neurons, local interneurons (LNs) and projection (output) neurons (PNs), most of which possess dense dendritic arbors confined to a single glomerulus [9]–[13]. PNs receive input from both olfactory receptor cells (ORCs) and LNs, and send output to higher olfactory centers via their long axons [11], [14], [15]. In contrast, the axonless LNs, which innervate many glomeruli, are mainly responsible for within-AL information processing [16]–[20]. The distinct morphological characteristics of PNs and LNs enabled us to identify these two types of neurons unambiguously with fluorescent dyes, satisfying the prerequisite for investigating the relationship among neuron types, spiking patterns and in the case of PNs, response characteristics. Further, PNs that innervate the moth's male-specific macroglomerular complex (MGC) respond selectively to components of the conspecific female sex pheromone [21], [22], allowing identification of these neurons based on their odor response properties.

Here we report that in the moth AL both PNs and LNs are spontaneously active, but these neuronal types have distinct firing patterns, with PNs producing randomly bursting spikes and LNs firing more regularly. Non-spiking LNs have been reported in other insects [17], [23], but still need to be confirmed in *M. sexta*. Incorporation of the firing patterns into a classification algorithm allowed us to distinguish PNs from LNs with up to 90% of accuracy. In addition, we found that spontaneous burstiness in MGC-PNs was positively correlated with spontaneous firing rate and with their responsiveness to pheromonal stimuli. Furthermore, pharmacological reduction of burstiness in the MGC-PNs resulted in a decrease of the neuron's response to odorant stimulation.

## Materials and Methods

### Intracellular recording and staining

*Manduca sexta* (L.) (Lepidoptera: Sphingidae), reared in the laboratory on an artificial diet, were used 1–3 days after adult emergence. We did not notice any apparent difference in the overall spiking patterns of day 1–3 animals. In the same species, Mercer and Hildebrand pointed out that bursty spike patterns are already formed at stage 6 (out of 18) of metamorphosis [24]. We postulate that spike patterns associated with different types of neurons are probably established in later developmental stages and persist through adult stage. Animals were dissected and prepared for intracellular recording according to established procedures [25]. Sharp microelectrodes were made from borosilicate glass capillaries with filament (1 mm outer diameter; 0.58 or 0.75 mm inner diameter; Sutter Instruments, Novato, CA) on a laser puller (P-2000; Sutter Instruments). The tip of the micropipette was filled with a 65 mM solution of Lucifer yellow CH (Sigma-Aldrich) in 200 mM LiCl, or with a solution of Alexa Fluor 568 hydrazide (10 mM in 200 mM KCl; Invitrogen, San Diego, CA). The shaft was filled with 2 M LiCl. The final electrode resistances ranged from 100 to 350 MΩ. Membrane potentials were amplified 10–50 fold using an amplifier (Axoclamp-2A, Molecular Devices, Foster City, CA; or IX2-700, Dagan Instruments, Minneapolis, Minnesota) in some cases coupled to a DC amplifier (LPF 202A, Warner Instruments, Hamden, CT) and digitized at 20 kHz (Digidata 1200 series Interface; Molecular Devices, Foster City, CA; some data were digitized using Datapack, Run Technologies, Mission Viejo, CA). To avoid voltage-dependence of spike patterns, no current was applied during recordings until dye injection. After physiological characterization, neurons were injected with either Lucifer yellow or Alexa 568 by passing hyperpolarizing current (0.3–1.5 nA) for at least 6 min. Upon completion of an experiment, the brain was excised and immersed in 2.5% formaldehyde fixative solution (pH 7.2) for at least 3 h, dehydrated through a graded series of ethanol solutions, and cleared with methyl salicylate (Sigma-Aldrich). Cleared brains were imaged as whole mounts (optical sections, 2 µm thick) with a laser-scanning confocal microscope (Nikon PCM 2000, Tokyo, Japan; or Carl Zeiss 510 Meta), equipped with a 457 nm argon laser and a 546 nm green HeNe laser.

### Juxtacellular recording, odor stimulation and pharmacology

Long-term recordings from single neurons, as required in the pharmacological experiments in this study, were achieved using the juxtacellular recording technique [26], [27]. In short, electrodes resembling those used for patch clamp recordings were pulled from thin-wall borosilicate glass capillaries (1 mm outer diameter; 0.78 mm inner diameter; Sutter Instruments, Novato, CA) using a laser puller (Sutter P-2000 , Sutter Instruments, Novato, CA), and filled with physiological saline, resulting in electrodes with <5 mΩ resistance. Electrodes were positioned in the MGC region of the AL and slowly advanced until a contact similar to that used for perforated-patch recordings was achieved. At this point, extracellular spikes could be distinguished from baseline noise. Signals were amplified 1000× times using an amplifier (Axoprobe-1A, Molecular Devices, Sunnyvale, CA) connected to a 10× DC amplifier (Model FC-23B, WPI, Sarasota, FL).

Olfactory stimuli were delivered to the preparation by injecting odor-laden air puffs onto a constant air flow (1 liter per minute) that was directed to the middle portion of the antenna ipsilateral to the AL from which recordings were made. Trains of 5 air puffs (50 ms each; 2 sec inter-pulse interval) from a glass syringe containing odors were generated using a solenoid-activated valve controlled by an electronic stimulator (WPI, Sarasota, FL). A piece of filter paper was placed in each glass syringe, bearing various amounts of a single pheromone component or a blend of the two key pheromone components (0.1–100 ng in decadic steps), or solvent alone. The stimulus compounds used were: (i) E10,Z12-hexadecadiennal (bombykal [Bal], the primary component of the conspecific female's sex pheromone) [28], [29]; (ii) E11,Z13-pentadecadiennal (“C15”, a chemically more stable mimic of another essential component of the sex pheromone) [30]; and (iii) a mixture of Bal and C15 (blend, 1∶1 ratio). Although we substituted C15 for the natural pheromone component, we refer to both Bal and C15 as pheromone components. MGC-PNs were characterized using the following physiological criteria: (1) response specificity to the pheromone components; and (2) multiphasic response pattern. In *M. sexta*, MGC-PNs have been extensively shown to produce predictable responses to the pheromone components according to the identity of the MGC glomerulus in which their dendrites arborize [10], [31]–[33]; MGC-PNs that innervate the Cumulus (one of the MGC glomeruli) are excited by antennal stimulation with C15 but inhibited (or not affected) by stimulation with Bal, whereas MGC-PNs that innervate the Toroid 1 (the second MGC glomerulus) are excited by stimulation with Bal but inhibited (or not affected) by stimulation with C15. Both types of MGC-PNs are excited by the blend (Bal+C15). MGC-PNs typically exhibit a triphasic (–/+/–) response pattern in intracellular recordings, i.e. a brief inhibitory response preceding a depolarization phase with spiking that is then followed by a period of delayed hyperpolarization and spike suppression. Juxtacellular recordings from MGC-PNs do not reliably show the initial inhibitory phase, but clearly display the excitatory response and the following inhibitory phase [27].

Bicuculline methiodide (Sigma-Aldrich, >95%) was diluted in physiological saline to a concentration of 25 µM (low dosage) or 500 µM (high dosage) and then bath-applied to moth preparations. This drug has been reported to act as a GABA_A_ receptor antagonist in *M. sexta* [34], [35] and an antagonist of small-conductance calcium dependent potassium channels (SK) in vertebrates [36].

### Detection and quantification of bursts

To describe the burstiness or the variation of interspike intervals (ISIs) of spike trains for different types of neurons, the Coefficient of variation (Cv), i.e. the standard deviation of ISIs divided by the mean of ISIs, was calculated from spontaneous traces (30–60 sec long). A second measurement of ISI variations, the Local Variation (Lv), was also calculated from these traces. Lv, which is less affected by the statistical non-stationarity of long spike trains [37], is defined as:
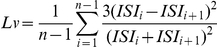
where *n* is the number of ISIs.

To quantify the spike-train burstiness more directly, we used the Poisson Surprise (S) criterion [38]. The S value for a given number of spikes within a time interval is a measure of the unlikelihood of observing that many spikes within that interval, i.e.
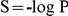
and
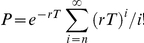
where *r* is mean firing rate and *T* represents the time interval.

The spikes are assumed to be generated randomly at an average rate and the time intervals between the occurrences of successive spikes are assumed to be independent of each other. This is the same as assuming that spikes come from a Poisson distribution with a certain rate, i.e. the mean rate of the spike train under investigation. The S value of n spikes within a time interval T is then the negative log of the probability of observing at least n spikes within T under a Poisson distribution of rate equal to the mean spiking rate.

**Figure 1:**
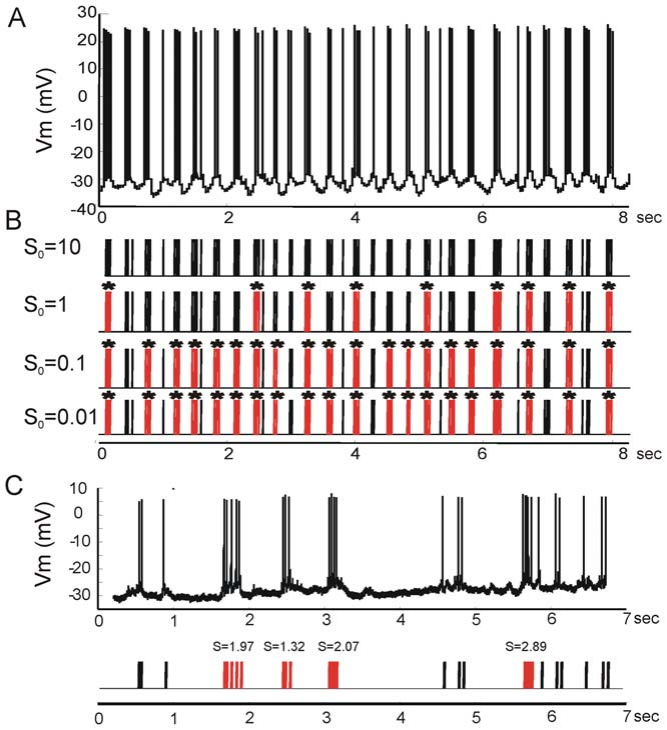
Burst detection. A rhythmically bursting trace was used to test the accuracy of the *Poisson Surprise* algorithm in detecting spike bursts (A). Each action potential (or spike) is represented by a tick mark in (B). With the decreasing initial *Surprise* value (S_0_) the successful detection rate increased from 0% to 88%. The detected bursts are marked with asterisks on top of the bursts. The same algorithm was also tested on non-rhythmically bursting traces using S_0_ = 0.1 (C), showing different *Surprise (S)* values calculated from four detected bursts. Higher *S* values appear to be associated with briefer bursts with high spiking frequency.

Using the Poisson Surprise method, a potential burst is detected by starting with a pair of successive spikes whose ISI is less than half the mean ISI of the entire spike train. Subsequent spikes are included if the ISI between successive spikes is less than the mean ISI. Poisson surprise values for the potential bursts (sets of spikes) are computed with the inclusion of each successive spike and the set with the highest Poisson surprise value is retained. The set is further refined by removing spikes at the beginning of the set if the surprise value is increased by doing so. Then the spike set is considered as a burst if its S value exceeds a predetermined threshold (S_0_) and if it contains a minimum of three spikes. The effectiveness of this algorithm is demonstrated in Fig. 1 (A–C), which shows a rhythmic bursting neuron recorded intracellularly (likely a PN with its soma in the anterior cluster of cell bodies [39]) and contains 24 apparent bursts in the 8 second period that was selected as a testing input for the algorithm (Fig. 1A). The accuracy of burst detection increased with decreasing initial values S_0_, but reached the maximum (88%) at S_0_ = 0.1 (Fig. 1B). This initial value was then used throughout the rest of analysis. A test on a randomly bursting PN demonstrated again the effectiveness of this algorithm (Fig. 1C).

### Classification and correlation analysis

The PN/LN burst parameters were entered into an internal classification algorithm (function: “Classify”, with “linear” option, Statistics toolbox) in Matlab (The Mathworks Inc, Natick, MA). For each run 20% of randomly selected PNs or LNs were used as the training group and the rest as the sample group. A percentage of correct classification was calculated from each run and the procedure was repeated 1000 times. To estimate the correct classification due to chance, the labels for the PNs and LNs were randomly shuffled. The new labels were checked against the original ones to calculate the percentage of labels that remained correct. This procedure was also repeated 1000 times.

Linear regression was performed to examine the correlation between the neuronal responsiveness (i.e. mean instantaneous firing rate during the response period) and the individual burst parameters. Subsequently, a Canonical Correlation Analysis (CCA) was performed to correlate the neuronal responsiveness and the integrated burstiness. Since CCA constructs canonical variates in such a way that the correlation between them is maximized, it is therefore important to assess the correlations produced by the procedure itself. To this end we randomly shuffled the response data thus breaking the pairings between the response data and the burst data. This new data matrix was then entered into CCA and the correlation coefficient was computed. This procedure was repeated 1000 times to calculate the magnitude of chance-induced correlation. Also, because each MGC-PNs was stimulated with 4 concentrations of the pheromone blend (from 0.1 ng to 100 ng in decadic steps), we obtained 4 replicates where the empirical correlation between the neuronal responsiveness and burstiness could be determined. MGC-PNs were chosen in this experiment because their specific ligands are known.

A similar correlation analysis was performed on spontaneous firing rate. Spike traces about 1 min long were extracted from each neuron. A cumulative curve was generated from the frequency distribution of ISIs of each trace, and the 5% increment point was identified on the curve. This point was then used as the cut-off threshold, which allowed selection of representative ISIs (85% of total) from each trace. Then the mean firing rate was calculated from these selected ISIs, which was subsequently correlated with the burst parameters of each neuron from which the ISIs were extracted.

## Results

Data reported here were collected from two series of experiments. First, we used intracellular recording and staining methods to sample the spontaneous activity of 81 PNs (63 PNs with arborizations in glomeruli other than the MGC and 18 MGC-PNs) and 85 LNs, all of which were morphologically identified (representative examples are shown in Fig. S1). The second series of experiments were focused on MGC-PNs (n = 12), in which we used a more stable method (juxtacellular recording) that allowed us to study the neuron's dose response and to pharmacologically manipulate their spontaneous spike patterns.

### PNs and LNs have distinct spontaneous spike patterns

**Figure 2:**
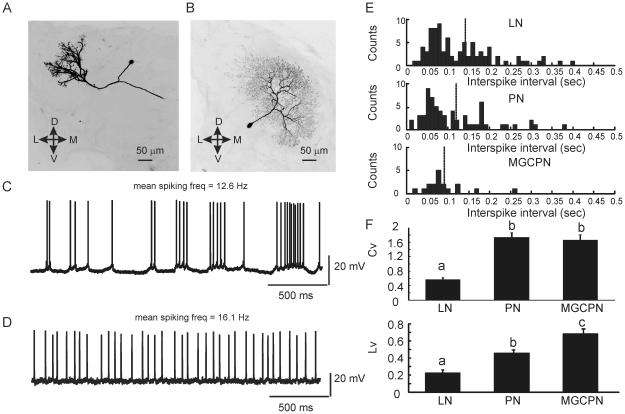
Distinct morphological and physiological properties of PNs and LNs. Shown in (A) and (B) are a PN innervating the Cumulus of the MGC and a LN arborizing in the medial portion of the AL. The MGC PN randomly produced spike bursts (C) whereas the LN fired tonically (D). The distribution histogram of interspike intervals (ISI) pooled from 85 LNs, 63 PNs and 18 MGC-PNs (each with 30–60 sec long spike trace) clearly shows a skewed distribution in these neurons (E). The vertical dashed lines indicate the averaged ISIs. Both Coefficient of Variation of ISIs (Cv) and Local Variation of ISIs (Lv) (Mean±SEM) show that LNs have significantly less variable ISIs comparing with that of PNs and MGC-PNs (F). Different letters indicate statistical significance (*Kruskal-Wallis H* test, *p*<0.01).

The spike traces of morphologically identified PNs, MGC-PNs and LNs were individually examined. Raster plots of spiking activities from these neurons revealed gross differences of their spike patterning. PNs, especially MGC-PNs, displayed more clusters of spikes whereas LN spikes were more evenly distributed (Fig. S2). The morphology of two such neurons is shown in Fig. 2A, B. The PN had dense arbors in the Cumulus (one of the MGC glomeruli), with its soma located in the medial cluster of neuronal cell bodies and the axon projecting to protocerebrum. The LN innervated the ventro-medial portion of the AL with its soma located in the lateral cluster of neuronal cell bodies. Both neurons were spontaneously active, but their spiking patterns were clearly different. The MGC-PN displayed bursting spikes with variable interspike intervals (ISIs) (Fig. 2C) whereas the LN produced a regular spiking pattern with rather consistent ISIs (Fig. 2D). Interestingly, when pooling all LNs the ISI histogram displayed a skewed distribution, with the major peak occurring at an ISI similar to that from PNs and MGC-PNs and the tail including larger ISIs (Fig. 2E). The mean ISI among the neuronal types was not statistically different (*Kruskal-Wallis H* test, *p*>0.05); however, the variance of ISI was significantly larger in PNs or MGC-PNs than in LNs (*Mann-Whitney U* tests, p<0.005 in both cases), suggesting a higher degree of variation in PN and MGC-PN spikes. A commonly used parameter for measuring variation, the coefficient of variation (Cv), was also calculated for each neuron (Fig. 2F, upper panel), revealing that PNs and MGC-PNs had significantly higher Cv values than LNs (*Kruskal-Wallis H* test, *p*<0.01). A third measure of spiking variability, the local variation (Lv), which is less affected by the statistical non-stationarity of long spike trains [37], again indicated that the spike pattern of PNs and MGC-PNs was significantly more variable than that of LNs (Fig. 2F, lower panel). This measure, however, revealed that the spike pattern of MGC-PNs was even more variable than that of the other uniglomerular PNs (*Kruskal-Wallis H* test, *p*<0.01).

**Figure 3:**
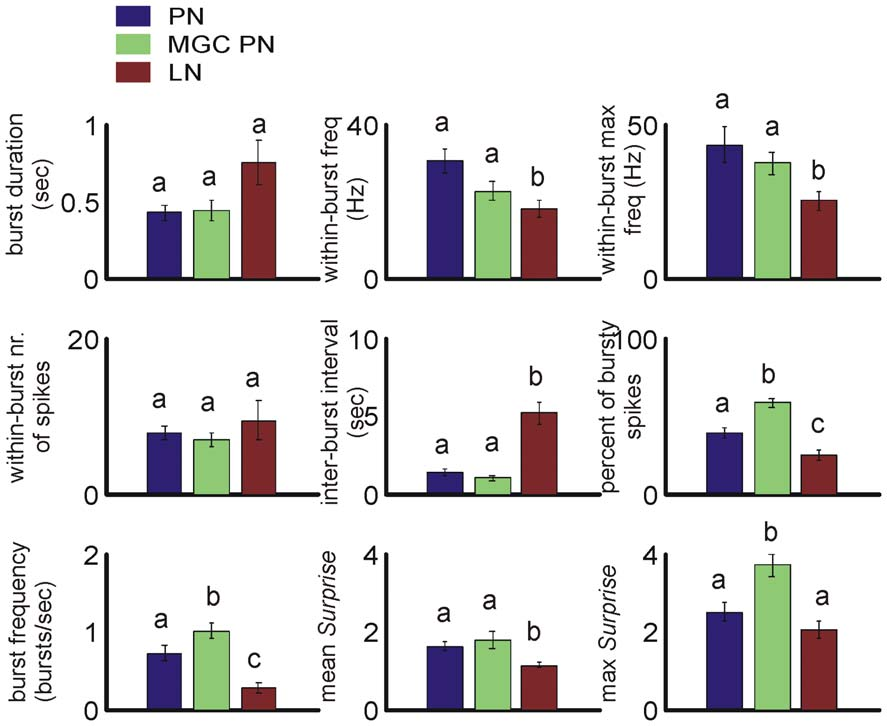
Burst quantification. Spike-train-burstiness is quantified with a set of parameters after the bursts are detected using *Poisson Surprise* algorithm from all three neuron types. Except for the parameters of “burst duration”, “within-burst number of spikes” and “max Surprise” all other parameters describing the burstiness of PNs (n = 63) or MGC-PNs (n = 18) are significantly different from that of LNs (n = 85) (*Kruskal-Wallis H*, *p*<0.05). Bar values are Mean ± SEM.

A possible source of spike variability comes from the burstiness of spike trains. We therefore applied a *Poisson Surprise* [38] algorithm to detect spike bursts on spike trains. Once detected, a burst was quantified with multiple parameters such as burst duration, within-burst spiking frequency, within-burst maximum spiking frequency, within-burst number of spikes, inter-burst interval, percentage of spikes that were included in bursts, mean burst frequency, mean *Surprise* value and maximum *Surprise* value. The same algorithm was performed on spike trains derived from PNs, MGC-PNs and LNs (Fig. 3). Results clearly show that LNs were significantly less bursting when compared with PNs and/or MGC-PNs, as shown by the above parameters. Additionally, MGC-PNs differed significantly from other uniglomerular PNs only in three parameters, i.e. the percentage of bursting spikes, the burst frequency and the maximum *Surprise* value, indicating a similar spiking pattern between these two groups of neurons, relative to the LNs. The differences revealed in these three parameters, along with the Lv measurement of spiking variation (Fig. 2F), suggest that the spiking activities of MGC-PNs could be even more variable. Although the trend was clear, the parameters in Fig. 3 were not equally informative. For example, LNs and PNs did not show statistical difference in the burst duration, the number of spikes within a burst, and the maximal Surprise value. Collectively, all these results indicate that the spontaneous spiking patterns of LNs and PNs significantly differ from each other, with PNs having higher within-burst spiking frequency, higher within-burst maximum spiking frequency, shorter inter-burst interval, higher bursting frequency and higher *Surprise* values (*Kruskal-Wallis* H test, *p*<0.05).

While the spiking activity of PNs was more variable or bursting than that of LNs, an overlap between these two types of neurons was also apparent. The Cv of PNs and LNs respectively ranged from ca. 0.1 to 2.3 and from 0.1 to 2.9, and the Cv of PNs was positively correlated with their Lv (R^2^ = 0.543, *p*<0.001) (Fig. S3A). However, more than 50% of PNs exhibited Cv>1 whereas only a few LNs displayed such high variability (Fig. S3B). The spiking variability was also evident in the auto-correlogram derived from PN spike traces. One PN with Cv = 0.24 displayed oscillatory autocorrelogram while another PN with a much higher Cv (2.05) totally lacked such oscillations (Fig. S3C).

Another interesting observation was that the bursts within a PN appeared to be similar (Fig. S4). In this example, the PN produced 270 bursts during a period of 8 min spontaneous activity. We examined the *difference* of two bursts that were either directly next to each other (Δ = 0, i.e. no other bursts in between), or with another burst in between (Δ = 1), or with two bursts in between (Δ = 2). In all three parameters examined – Poisson surprise (S) (Fig. S4 A–C), within-burst spiking frequency (Fig. S4 D–F) and within-burst number of spikes (Fig S4 G–I) – the distribution histograms were bell-curve shaped with its center at *difference = 0*, suggesting the two bursts were alike. We compared bursts with Δ value up to 25, finding that the bell-curve-shaped histograms were maintained.

### Statistical classification of neuron types

**Figure 4:**
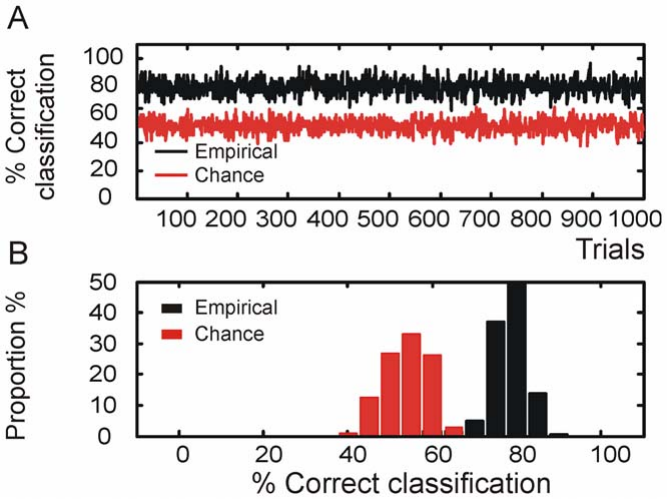
Statistical classifications of neuron types based on the spike-train's burstiness. A linear classification process, repeated 1000 times with each time having a randomly chosen set of training group, yields up to 90% of accuracy in distinguishing the PNs from LNs (black line); the rate of correct classification by chance is 50% (red line) (A). Histogram data show that the empirical and chance classification has about 80% and 50% of accuracy, respectively (B).

Next we tested if the bursting features could be used to statistically distinguish PNs from LNs. Because identification of MGC-PNs can be based on their unique responsiveness to the sex pheromone, this group of cells was not included in the classification analysis. Nine parameters describing the LNs' and PNs' burstiness along with their categorical labeling were entered into a linear classification algorithm (Materials and Methods), and then the correct classification percentage was calculated based on the output of the algorithm and the original data. This procedure was repeated 1000 times, randomizing the order of neurons each time. We found that on average the success rate was about 80% with a maximal accuracy of 90% (Fig. 4 A, B).

### Correlation between burstiness and response firing rate

**Figure 5:**
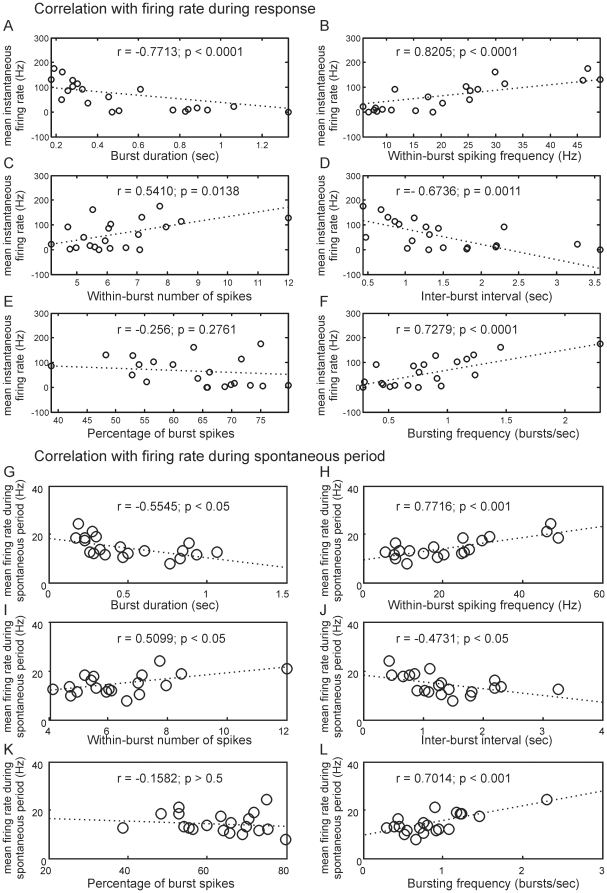
Correlation between MGC-PNs' burstiness and firing rates during odor-evoked response and spontaneous (non-stimulation) periods. Burst parameters (measured during spontaneous period) were used in a linear regression analysis. During response period, negative correlations were found for burst duration, inter-burst interval and percentage of burst spikes; positive correlations were found for within-burst spiking frequency, within-burst number of spikes and bursting frequency (A–F). During spontaneous period, similar correlations were also found between the burst parameters and firing rate (G–L).

Next we examined the correlation between MGC-PNs' mean instantaneous firing rate – firing frequency calculated from the inverse of inter-spike intervals - during odor response (to 50 ms blend 10 ng stimulation) and their burst parameters during spontaneous (non-stimulation) period (Fig. 5 A–F). We found that the response firing rate was positively correlated with the within-burst spiking frequency (*r* = 0.82, *p*<0.0001), the within-burst number of spikes (*r* = 0.54, *p* = 0.0138) and the bursting frequency (*r* = 0.73, *p*<0.0001) (Fig. 5 B, C, F), but negatively correlated with the burst duration (*r* = −0.77, *p*<0.0001) and the inter-burst interval (*r* = −0.67, *p* = 0.0011) (Fig. 5 A, D).

In MGC-PNs, the odor-evoked responses essentially consist of large burst of spikes. If the same mechanisms underlie the production of spontaneous bursts and odor-evoked responses, one would expect a positive correlation between the burst parameters and the spontaneous firing rate. This was indeed the case (Fig. 5 G–L).

**Figure 6:**
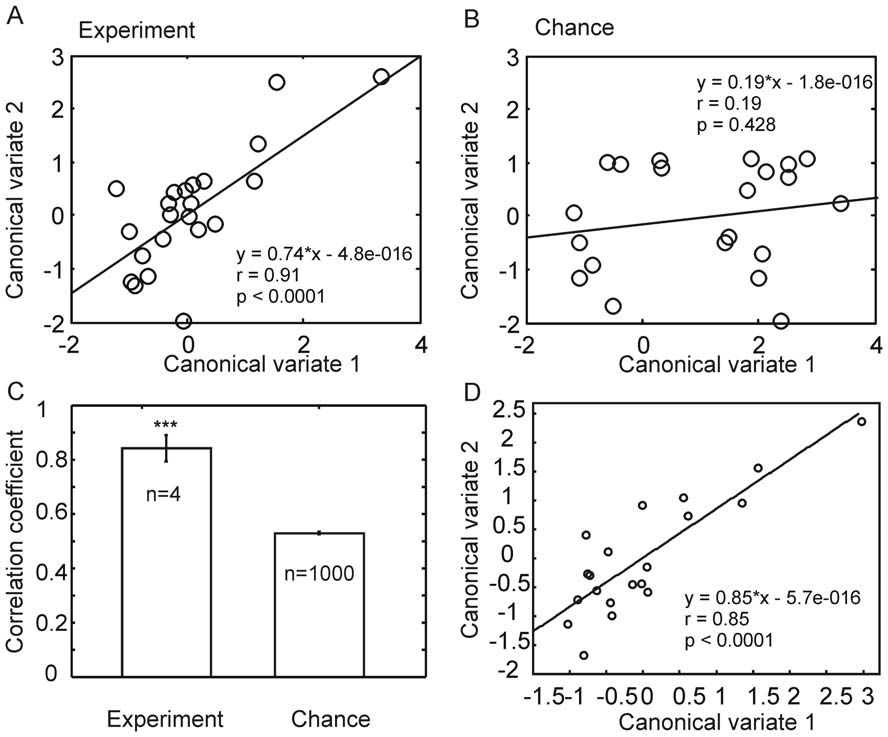
Results from the Canonical Correlation Analysis (CCA) showing significantly positive correlations between the MGC-PNs' burstiness and their firing rate during response and spontaneous periods. CCA linearly combines individual parameters to generate Canonical variates (Variate 1 for burstiness and Variate 2 for responsiveness) that satisfy the condition that the correlation between Variate 1 and Variate 2 is maximal (A). As a control for the procedure itself, CCA was performed on the data where the order of the neurons was randomly shuffled for the dependent variate (thus breaking the pairing between the burstiness and responsiveness). The correlation coefficient so obtained was not significant (B). The shuffling procedure was repeated 1000 times and the results show that chance-induced correlation is significantly lower than the correlation obtained in experiments where 4 odor concentrations ranging from 0.1 ng to 100 ng in decadal steps were used (C, Mann-Whitney U test, p<0.0001). CCA also revealed a significant positive correlation between the two canonical variates representing burstiness and spontaneous firing rate (D).

We next minimized the correlation redundancy due to the relatedness of burst parameters. For this, we conducted the Canonical Correlation Analysis (CCA) (Materials and Methods), which linearly combined all burst parameters after weighing the contribution of each one to generate a Canonical Variate (Canonical variate 1 in Fig. 6 A), and then correlated it with the response firing rate (Canonical variate 2 in Fig. 6 A). The correlation between these two variates was highly significant (*r* = 0.91, *p*<0.0001), indicating that the MGC-PNs' bursting property was predictive of their responsiveness – the higher the burstiness, the stronger the response. As a control we also conducted the CCA after randomly shuffling the order of these neurons, thus breaking the pairing between the firing rate and the burst parameters. The correlation so obtained was not significant (*r* = 0.19, *p* = 0.428) (Fig. 6 B), thus supporting that the positive correlation shown in Fig. 6 A was not due to the CCA procedure itself. Furthermore, the random shuffling procedure was repeated 1000 times, resulting in correlation coefficients (0.53±0.12; mean ± Std; n = 1000) that were significantly lower than the correlation coefficients (0.84±0.095; mean ± Std; n = 4) derived from the experimental data (*Mann-Whitney U* test, *p*<0.0001) (Fig. 6 C). The latter preserved the paired relationship between the neurons' burstiness and the mean instantaneous firing rate evoked by stimulation with different odor concentrations (0.01 ng, 0.1 ng, 1 ng, 10 ng) (n = 4). Additionally, we performed CCA on spontaneous traces and found that the MGC-PNs' burstiness was also positively correlated with their spontaneous firing rate (Fig. 6 D, *r* = 0.85, *p*<0.0001).

### Pharmacological manipulation of MGC-PNs' burstiness

**Figure 7:**
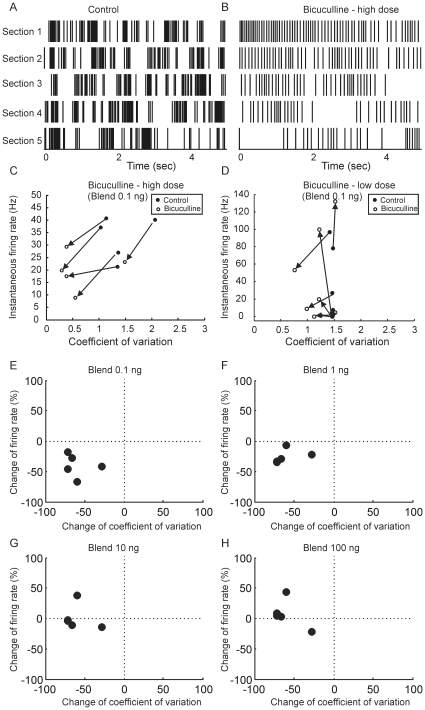
Pharmacological manipulations of the neuronal burstiness and the effects on responsiveness to odors. Application of bicuculline at 500 µM apparently caused an MGC-PN to change its bursting spontaneous activity to a tonic pattern (A, B). Tick marks represent spikes, and the vertically arranged rows are continuous in time. The coefficient of variation (Cv) and instantaneous firing rate during the response of MGC-PNs (n = 5) to blend (0.1 ng) were measured before (open circles) and during (filled circles) the bath application of bicuculline methiodide (high dose – 500 µM) (C). Arrows point at the direction of changes, showing that under the drug application every neuron reduces its Cv (thus its burstiness) (Mann-Whiteney U test, p<0.05) and its response firing rate. This effect, however, is drug-dosage dependent, as shown by inconsistent changes of Cv and response firing rate under low dose treatment (25 µM, n = 7) (D). Furthermore, the drug effect on response firing rate is also dependent on odor concentrations. While bicuculline reduces Cv in a magnitude of 25%∼75% across 4 odor concentrations (blend 0.1 ng–100 ng in decadal steps), its effect on reducing the response firing rate is weakened and even reversed with increasing odor concentrations (E–H).

Previous studies showed that bicuculline methiodide produced changes in spontaneous firing patterns [34], [40], including one study which showed that MGC-PNs changed from a randomly bursting to a tonic firing pattern during drug bath application [27]. We thus took advantage of this pharmacological tool to investigate the link between the MGC-PNs' burstiness and their odor responsiveness. In order to quantify how this pharmacological intervention affects spike burstiness, the coefficient of variation (Cv) for the interspike intervals was measured during a 1-minute recording of spontaneous activity before and during bicuculline application. Similarly, the odor-evoked mean instantaneous firing rate was also calculated before and during the drug application. As a result of high-dose (500 µM) bicuculline superfusion, MGC-PNs (n = 5) ubiquitously decreased their Cv as well as their firing rate (Fig. 7 C). An example is shown in Fig. 7 A–B, where the bursting spontaneous activity of an MGC-PN was apparently changed to a more regular pattern due to high-dose bicuculline application. The reduction of Cv and firing rate was not consistently observed when neurons were perfused with a low dosage of bicuculline (25 µM) (n = 7) (Fig. 7 D). Furthermore, the drug effect was also dependent on odor concentration. Under the same high-dosage application of bicuculline, MGC-PNs reduced their Cv by about 50%, but the modulation on the response firing rate varied with odor concentration (blend 0.1 ng–100 ng in decadic steps). The largest reduction (ca. 50%) on firing rate was observed upon stimulation with 0.1 ng of the blend (Fig. 7 E), but this trend became less obvious at a higher concentration (1 ng) (Fig. 7 F), and even reversed at 10 ng (Fig. 6G) and 100 ng (Fig. 7 H).

## Discussion

Neural circuits are composed of different types of neurons, specifically wired to accomplish various physiological functions. Although action potentials (or spikes) are generally considered as the currency of information transmission in nervous systems, the more complex spiking patterns generated by different types of neurons may differ drastically according to their biophysical properties. These properties may facilitate specific functions of certain neuron types in neural circuits. In the olfactory bulb, the external tufted cells produce spike bursts at theta frequencies whereas the other types of juxtaglomerular neurons rarely produce spikes [3], [7]. In the insect AL, the morphological differences between PNs and LNs are clear, the former having a long axon exiting the AL and the latter being axonless. Reports on the differences of their spontaneous firing patterns, on the other hand, are rather scattered in the literature. For example, rhythmic bursts as well as tonic spiking patterns were reported in LNs in the moth *M. sexta* [16] and *Drosophila* [18]. Bursting patterns were found in both PNs and LNs in *Agrotis segetum* [41]. In the AL of honeybees, PNs innervating multiple glomeruli produce rhythmic bursts, but LNs generate irregular bursts of spikes [42]. A recent study revealed two physiologically distinct types of LNs in the AL of cockroaches - Type I being capable of generating sodium spikes and Type II producing calcium spikes only [43]. Two other studies took advantage of the presence of specific GAL-4 lines in *D. melanogaster* to study the morphological and electrophysiological diversity of AL LNs [18], [20]. In [18], two classes of LNs, Krasaviez_class1 and Krasaviez_class2, were shown to generate tonic spike pattern when responding to input currents whereas other two classes, NP1227_class1 and NP2426_class1 LNs, did not process the input stimulus continuously but rather responded transiently. The panglomerular LNs, as described in [20], had higher background firing rate and weaker change of firing rate in response to odor stimuli, compared with other LNs that have more heterogeneous innervation patterns. All these data clearly demonstrate the diversity of spiking patterns in AL neurons.

In this study we analyzed a large sample of morphologically identified PNs (n = 63), MGC-PNs (n = 18) and LNs (n = 84) in the AL of *M. sexta*. Although we did not find a clear-cut boundary that separates the firing patterns of PNs from that of LNs, the PNs tended to produce randomly bursting spikes with more variable interspike intervals (ISI), which is reflected in the larger coefficients of variations of ISI and also by the differences in burst parameters (Fig. 2; Fig. 2; Fig. S3). In contrast, the firing pattern of LNs appeared to be more regular or tonic. Based on the firing pattern characteristics, up to 90% of neurons were successfully classified as PNs or LNs (Fig. 4), corroborating the idea that the differences between the firing patterns of these two neuronal populations may be related to their functions in AL circuitry. PNs are responsible for transmitting information to third order olfactory neurons in the protocerebrum. In comparison with single spikes, a burst of spikes at high frequency may be advantageous in transmitting information [44]. LNs, on the other hand, as critical elements in modulating local circuits, may use tonic release of neural modulators to regulate global properties such as sensitivity and gain control. In *D. melanogaster*, wide-field LNs showed spike suppression and weak changes in firing rate in response to odor stimulation, and because they are mostly GABAergic, it was proposed that odor-evoke activities in these LNs dis-inhibit the entire AL [20]. It is worthwhile noting if spike patterns are related to how transmitters are released. Our data suggest that transient (or bursting) and tonic releasing may be two major mechanisms by which PNs and LNs respectively release transmitters. Further investigations are required to address whether bursting and tonic patterns are inter-changeable within a neuron (and if so, what are the mediating factors) or are permanently associated with certain neurons. The pharmacological experiments in this and our previous study (Fig. 7, [27]) showed that bicuculline methiodide caused MGC-PNs to change their spontaneous spiking patterns from randomly bursting to tonic pattern, and also change their odor-evoked responses from bursting to long lasting pattern. These results suggest that GABA-A receptors and/or small-conductance calcium-activated potassium channels (SK) [36] may underlie the transformation of spiking patterns. Verification of SK channels and their functions in AL neurons has yet to be determined, but in vertebrates, SK channels play important roles in shaping spiking patterns [45], [46].

Our results also demonstrate that the bursting characteristics of MGC-PNs are correlated with their responsiveness to pheromones – the higher degree of burstiness the stronger response (Fig. 5, 6). Pharmacologically reducing the neurons' burstiness resulted in decreased response intensity, although this phenomenon was odor concentration dependent (Fig. 7). These results do not indicate a causal relationship between burstiness and response intensity, but suggest that common mechanisms may underlie both phenomena. Another possibility is that the burstiness of PNs may be caused by input from olfactory receptor cells (ORCs). In lobsters [2], ORCs produce rhythmic bursts. ORCs from pheromone-responsive sensilla in *M. sexta*, however, produce random bursts [47]. We argue that the periphery burstiness might not directly determine the bursting characteristics of PNs because if that were the case, the extensive convergence of ORCs onto PNs would most likely have transformed the random bursting pattern to other patterns such as long lasting firing. Furthermore, in developing moth ALs, experimental evidence indicates that the influence of peripheral neurons on the spiking patterns of AL is minimal [24]. This may not be the case in adult moths, however. Future deafferentation experiments should shed more lights on the origin of spiking patterns of AL neurons in adult moths.

In addition to the correlation between burstiness and responsiveness, our analysis also revealed that the burstiness of MGC-PNs is positively correlated with their spontaneous firing rate (Fig. 5, 6). In other words, bursting neurons tend to fire spikes at high frequency, either with or without stimulus. This result suggests that an overlapping set of conductances may be activated during the production of spontaneous bursts as well as during odor responses, but characterization of these conductances is beyond the scope of this study. Our observation that two categories of spiking pattern occur in AL neurons is consistent with earlier studies in the same species [24], [48], where putative PNs and LNs showed marked difference in the occurrence of sodium spikes.

Neuronal bursts are transmitted across synapses more reliably than isolated spikes [44]. In the natural environment of moths, an odor stimulus can become extremely low in concentration when it is far away from the odor source. Neuronal burstiness may thus facilitate detecting and processing such weak stimuli. In the moth AL, individual PNs do not produce spike bursts to the same extent; instead, they exhibit a range of burstiness, as measured by the coefficient of variation (Fig. S3). This may reflect the functional differentiation among PNs, with some neurons being more sensitive and some being less sensitive to olfactory stimulation. As a population, these neurons offer a full dynamic range to encode naturally fluctuating odor stimuli.

## Supporting Information

Figure S1 **Examples of spike traces and morphology of PNs and LNs.** The spike traces from 5 LNs and 5 PNs are shown in (A), demonstrating the variation of their spontaneous spike patterns. The overall tonic pattern in LNs and bursting patterns in PNs are apparent. The morphological characteristics of two neurons (A9, A10) are shown in (B).
(TIF)

Figure S2 **Raster plots showing 3 seconds of spontaneous spiking activities from MGC PNs (n = 18), PNs (n = 63) and LNs (n = 85).** Rows are arranged in ascending order of Cv values.
(TIF)

Figure S3 **PNs and LNs produce spike patterns of distinct but overlapped temporal features.** Linear regression analysis on interspike intervals reveals a significant positive correlation between the two measures of spiking variations (or burstiness) – the Coefficient of variation (Cv) and the Local variation (Lv) (A). Although overlapping, the Cv values of most LNs are below 1 while more than 50% of PNs and MGC PNs have Cv higher than 1, indicating more variability in PN and MGC PN's spontaneous spiking pattern (B). The relationship between Cv and spiking pattern is also evident from the autocorrelograms of spike traces. Two PNs were selected (asterisks in the middle panel of B), one with a Cv of 2.05 and the other 0.24. The former shows no regularity (thus highly variable) and the latter displays apparent oscillations, suggesting a regular spike pattern (C).
(TIF)

Figure S4 **Bursts are similar within a PN.** The difference between two bursts, which were directly next to each other (Δ = 0), or with another burst in between (Δ = 1), or with two other bursts in between (Δ = 2), was calculated by subtracting the Poisson surprise (S) (A–C), within-burst spiking frequency (D–F) or within-burst number of spikes (G–I) of one burst from that of the other. The relative proportion of these difference values was shown in frequency distribution histograms, which appear to be bell-curve shaped in all conditions. Difference value of zero (or close to zero) indicates two bursts are identical or similar.
(TIF)
