# Supplementary figures and images for: Spiking Patterns and Their Functional Implications in the Antennal Lobe of the Tobacco Hornworm Manduca sexta
Source: PLoS One. 2011 Aug 29;6(8):e23382. doi: 10.1371/journal.pone.0023382 (PMC3163580; doi:10.1371/journal.pone.0023382)

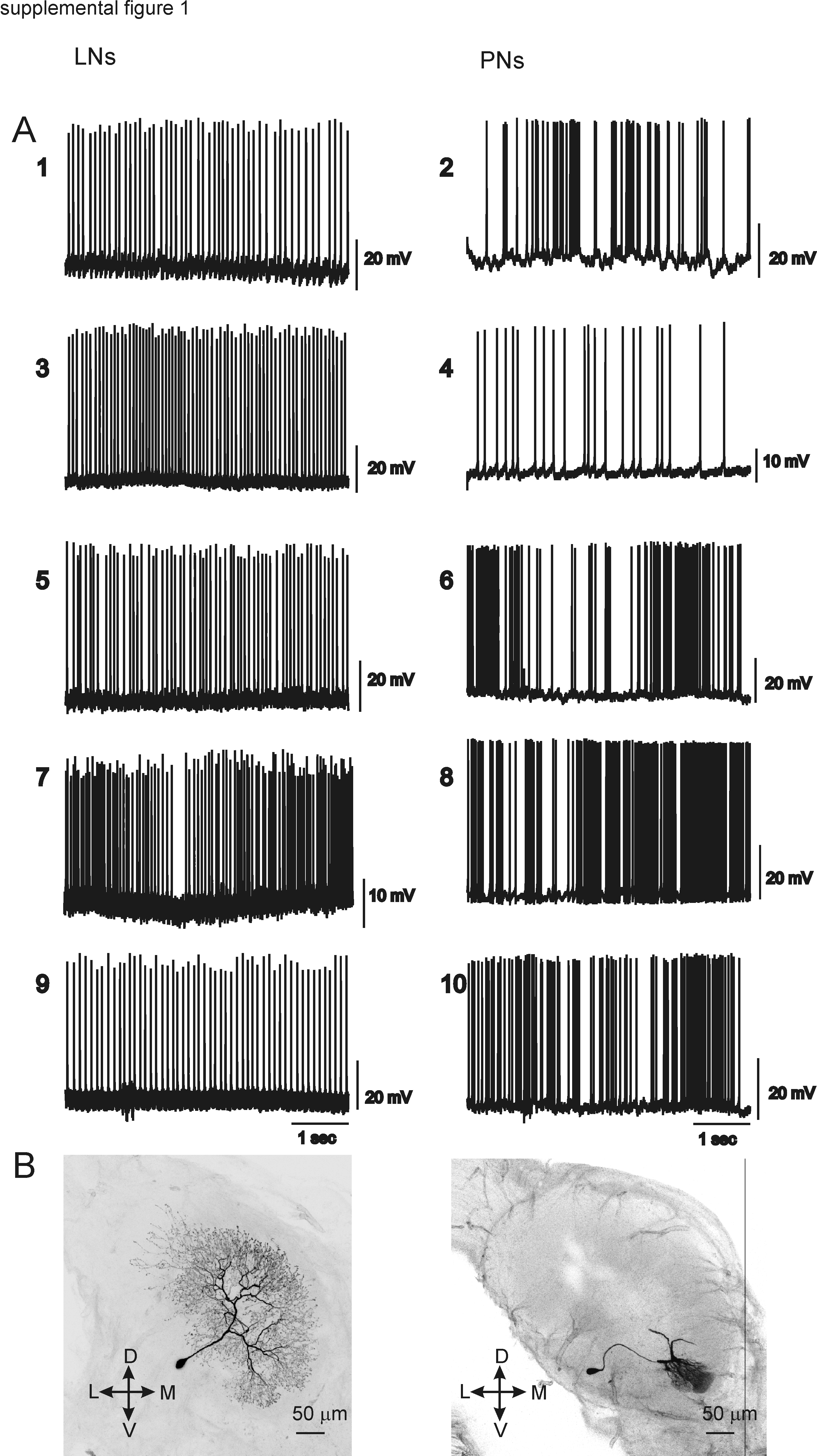

Supplement: Figure S1 — Examples of spike traces and morphology of PNs and LNs. The spike traces from 5 LNs and 5 PNs are shown in (A), demonstrating the variation of their spontaneous spike patterns. The overall tonic pattern in LNs and bursting patterns in PNs are apparent. The morphological characteristics of two neurons (A9, A10) are shown in (B). (TIF) [file pone.0023382.s001.tif]

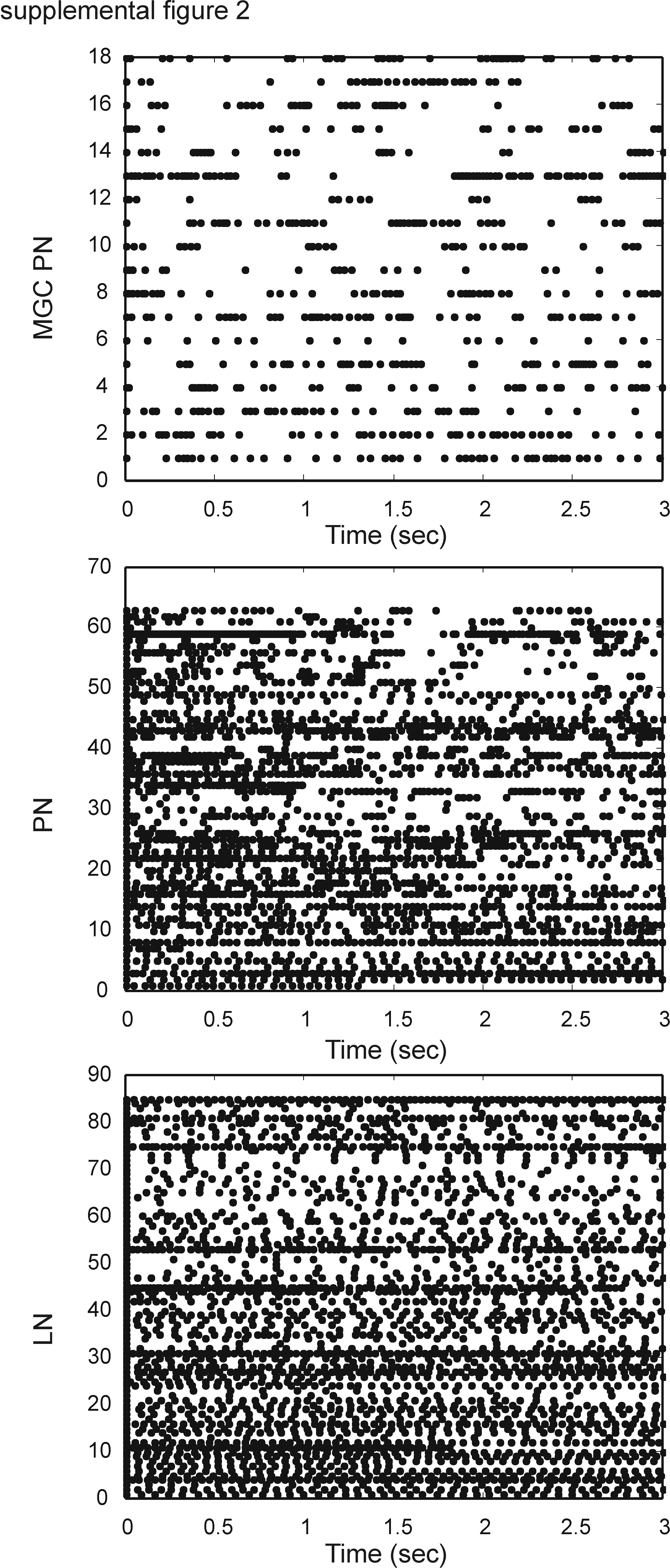

Supplement: Figure S2 — Raster plots showing 3 seconds of spontaneous spiking activities from MGC PNs (n = 18), PNs (n = 63) and LNs (n = 85). Rows are arranged in ascending order of Cv values. (TIF) [file pone.0023382.s002.tif]

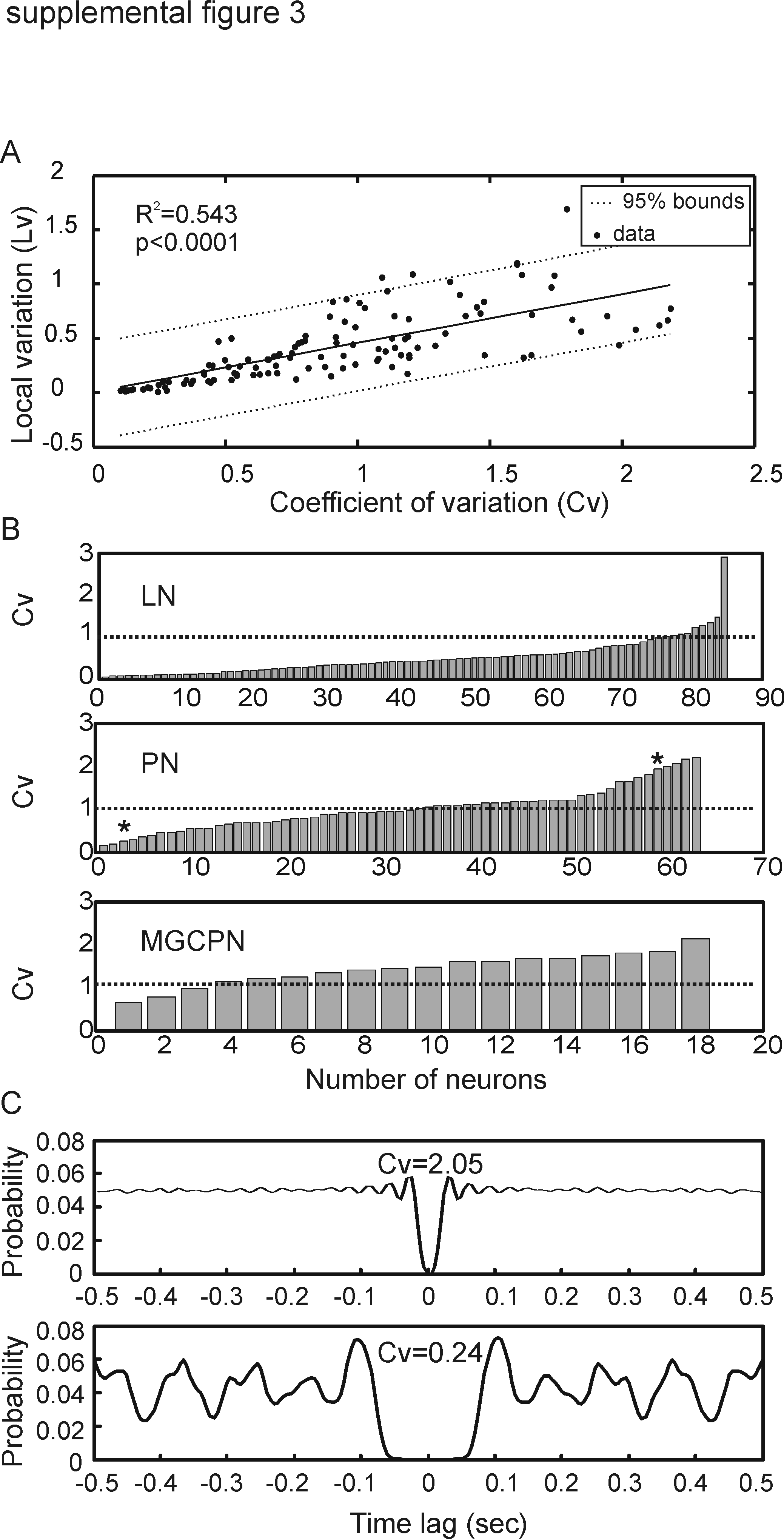

Supplement: Figure S3 — PNs and LNs produce spike patterns of distinct but overlapped temporal features. Linear regression analysis on interspike intervals reveals a significant positive correlation between the two measures of spiking variations (or burstiness) – the Coefficient of variation (Cv) and the Local variation (Lv) (A). Although overlapping, the Cv values of most LNs are below 1 while more than 50% of PNs and MGC PNs have Cv higher than 1, indicating more variability in PN and MGC PN's spontaneous spiking pattern (B). The relationship between Cv and spiking pattern is also evident from the autocorrelograms of spike traces. Two PNs were selected (asterisks in the middle panel of B), one with a Cv of 2.05 and the other 0.24. The former shows no regularity (thus highly variable) and the latter displays apparent oscillations, suggesting a regular spike pattern (C). (TIF) [file pone.0023382.s003.tif]

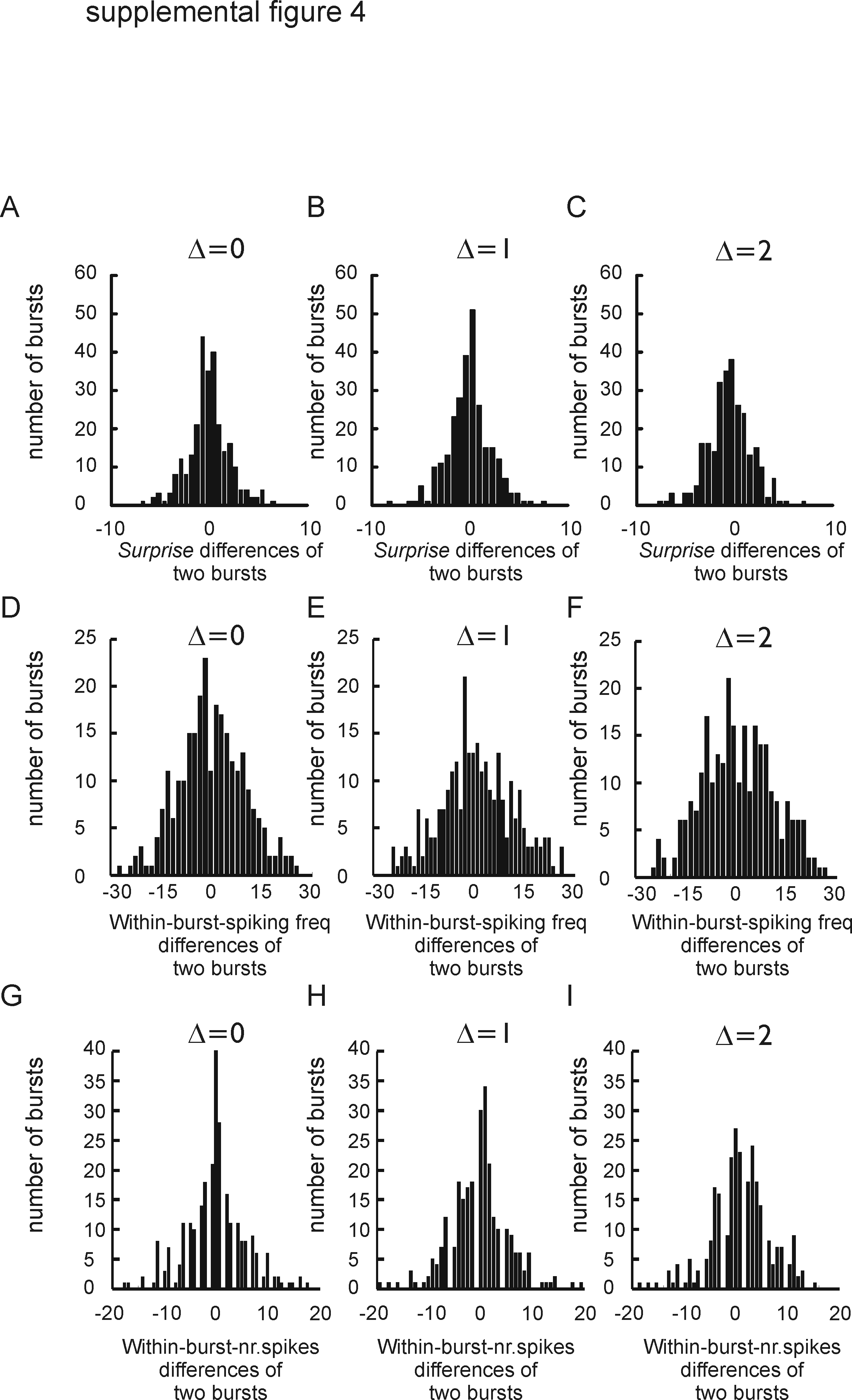

Supplement: Figure S4 — Bursts are similar within a PN. The difference between two bursts, which were directly next to each other (Δ = 0), or with another burst in between (Δ = 1), or with two other bursts in between (Δ = 2), was calculated by subtracting the Poisson surprise (S) (A–C), within-burst spiking frequency (D–F) or within-burst number of spikes (G–I) of one burst from that of the other. The relative proportion of these difference values was shown in frequency distribution histograms, which appear to be bell-curve shaped in all conditions. Difference value of zero (or close to zero) indicates two bursts are identical or similar. (TIF) [file pone.0023382.s004.tif]
